# Asthma: A Loss of Post-natal Homeostatic Control of Airways Smooth Muscle With Regression Toward a Pre-natal State

**DOI:** 10.3389/fped.2020.00095

**Published:** 2020-04-16

**Authors:** Michael B. Anthracopoulos, Mark L. Everard

**Affiliations:** ^1^Respiratory Unit, Department of Paediatrics, University of Patras, Patras, Greece; ^2^Division of Paediatrics & Child Health, Perth Children's Hospital, University of Western Australia, Perth, WA, Australia

**Keywords:** Asthma, airways smooth muscle, homeostasis, poor control, exacerbations, evolution, research direction

## Abstract

The defining feature of asthma is loss of normal post-natal homeostatic control of airways smooth muscle (ASM). This is the key feature that distinguishes asthma from all other forms of respiratory disease. Failure to focus on impaired ASM homeostasis largely explains our failure to find a cure and contributes to the widespread excessive morbidity associated with the condition despite the presence of effective therapies. The mechanisms responsible for destabilizing the normal tight control of ASM and hence airways caliber in post-natal life are unknown but it is clear that atopic inflammation is neither necessary nor sufficient. Loss of homeostasis results in excessive ASM contraction which, in those with *poor control*, is manifest by variations in airflow resistance over *short* periods of time. During viral *exacerbations*, the ability to respond to bronchodilators is partially or almost completely lost, resulting in ASM being “locked down” in a contracted state. Corticosteroids appear to restore normal or near normal homeostasis in those with poor control and restore bronchodilator responsiveness during exacerbations. The mechanism of action of corticosteroids is unknown and the assumption that their action is solely due to “anti-inflammatory” effects needs to be challenged. ASM, in evolutionary terms, dates to the earliest land dwelling creatures that required muscle to empty primitive lungs. ASM appears very early in embryonic development and active peristalsis is essential for the formation of the lungs. However, in post-natal life its only role appears to be to maintain airways in a configuration that minimizes resistance to airflow and dead space. In health, significant constriction is actively prevented, presumably through classic negative feedback loops. Disruption of this robust homeostatic control can develop at any age and results in asthma. In order to develop a cure, we need to move from our current focus on immunology and inflammatory pathways to work that will lead to an understanding of the mechanisms that contribute to ASM stability in health and how this is disrupted to cause asthma. This requires a radical change in the focus of most of “asthma research.”

“*When I use a word*,” Humpty Dumpty said in a rather scornful tone, “*it means just what I choose it to mean – neither more nor less*.”

*Through the Looking Glass Lewis Carroll 1871*

No disease was less understood by medical men, than asthma. Every difficulty of breathing, if fixed and continuous, was designated asthmatic; and the same indefinite application of the term still remains in vulgar use. This general application of the word caused it to be employed to denote a variety of morbid states of the lung, very different from one another.

*A practical treatise on the principal diseases of the lungs, GH Weatherhead. 1837*

**Homeostasis** is the property of a system within an organism in which a variable is actively regulated to remain very nearly constant.

## Introduction

It is now 145 years since Dr. Theodore Williams wrote about the pathology and treatment of spasmodic asthma (1). The contents of his article (summarized in Table 1) probably reflects the understanding of most doctors in the twenty-first century (and indeed demonstrates greater insight than most current medical practitioners) with the only notable omission being the dramatic impact of corticosteroids and the recognition that selective β2-agonists can provide rapid and symptomatic relief. He noted the hereditary predisposition; the importance of muscle spasm; the tenacious secretions and thickened airways walls; the importance of catarrhal infection in producing “spasmodic” asthma [viral exacerbations]; the role of environmental factors especially pollens, dust, and changes in weather; the value of avoiding precipitating factors, the presence of inflammation, the reversible hyperinflation, the refractory period, the patient's experience of greater difficulty breathing in than breathing out (2) and the value of bronchodilators.

**Table 1:** Summary of Williams, 1874 (1).

Hereditary predisposition plays a part
Muscle spasm is a central component of the disease
Tenacious secretions and thickened walls
Catarrhal attacks commonly cause “spasmodic” asthma
*[viral induced exacerbations-respond poorly to bronchodilators]*
Environmental precipitants
pollens, dust, chemicals, cold air etc.
May also be triggered by emotion, indigestion
Infection may be one of the causes
[“but be careful as almost everything in this country is attributed to a cold”]
Different patterns in different subjects
Typically causes cough, wheeze & difficulty breathing
Pts find it more difficult to breath in than out during an episode
Reversible emphysema *(hyperinflation)*
Describes the refractory period and effect of a deep breath
Commonly co-exists with eczema
Avoidance of triggers such as hay is helpful
Smoky cities are good for asthma for people from country areas
Bronchodilators are helpful
Patients often know as much about treating their disease as doctors
“it is, happily, not a fatal disease, yet it is one which gives rise to a large amount of suffering”
Can be associated with hysteria
*[dysfunctional breathing]*

A search on PubMed indicates that more than 185,000 articles, at a current rate of more than 21 papers a day, have been published on the topic of asthma since Dr. Williams' article. Despite the billions of dollars and countless hours expended in generating this body of work, we still cannot define the key component that underlies this condition and appear no nearer to finding a cure.

This inability to define the condition is associated with a depressingly high on-going prevalence of mis-diagnosis (both over and under) (3–5), morbidity and mortality in most countries. The introduction of inhaled corticosteroids (ICS) in the early 1970s (6, 7) was the one great step forward in the intervening 140 years; yet despite having safe and effective therapy, levels of morbidity and mortality remain unacceptably high, particularly in countries such as the U.K. and Australia^1^ (8). This is largely because doctors do not appear to do the simple things well. The failure to correctly identify the key component of the condition probably plays a central role in the respiratory fraternity's failure to impact on care throughout the community.

## Fashions in the Definition of Asthma

Dr. Whitehead's observation 182 years ago that “*No disease was less understood by medical men, than asthma”* (9) still resonates today. The on-going struggle to develop an agreed definition was recently reviewed in part by Hargreaves and Nair (10). The potentially central role for airways smooth muscle (ASM) in the causation of asthma was first established during the C19th starting with the great Rene Laennec who noted “*It is well understood that the spasmodic contraction of these fibers may be far enough to control the air ducts and to prevent the penetration of air into a large part of the lungs”* (11). Williams noted that “*Laennec's theory of asthma, was that the attack depended on spasm of the bronchial muscles”* which had recently been described by Reisseisen and observed by Laennec (1, 11). The functional activity of the ASM was confirmed by the middle of the century by Dr. Williams 's father (12) and others. In the USA, William Osler was echoing these views noting that excessive ASM contraction is the key feature that characterizes asthma (13). These pioneers recognized that the disease was generally associated with inflammation but emphasized the central, fundamental role played by excessive ASM constriction.

At around the turn of the century, the description of “anaphylaxis” and “allergic” responses propagated a belief that asthma was an “allergic” disease often being linked to other conditions then believed to be a consequence of “hypersensitivity” such as epilepsy and migraines. In its original sense, “allergy” referred to a change in the host in response to exposure to an environmental agent(s) (infective or otherwise), which could be both protective, as in limiting the impact of being re-exposed to an infectious agent (immunity) and detrimental, should the response elicited cause harm (hypersensitivity) (14). The idea that asthma was primarily a “neurological condition” arose in the C19th from the studies of Dr. Williams Sr. and others who demonstrated that ASM contracted in response to electrical stimulation. These ideas were often merged into a model in which a “sensitivity” or “allergy” to certain stimuli (infectious and non-infectious) drove a neurological response mediated via the vagus and parasympathetic system that caused ASM to constrict (15, 16). Hence, for much of the C20th asthma was viewed as a condition caused by ASM constriction driven by neurological stimuli as part of a “hypersensitivity reaction.”

**Figure 1:**
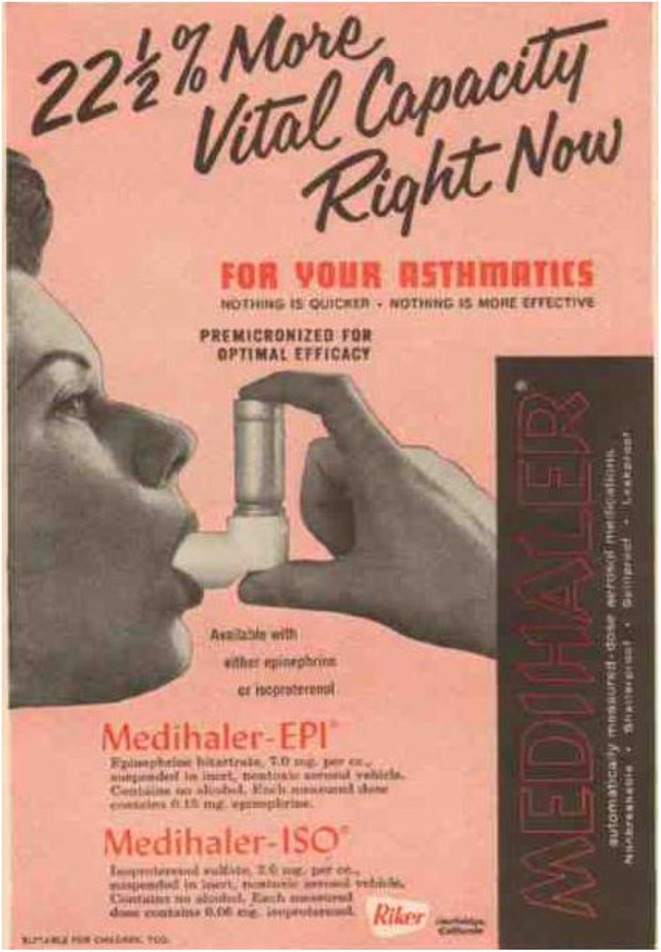
Advert for Medihaler (1956) http://museum.aarc.org/gallery/asthma-management/.

In the early 1960s Scadding and others proposed that asthma is a “*disease characterised by wide variation over short periods of time in resistance to flow in intrapulmonary airways*.” This was widely accepted and adopted by the ATS (17). This placed bronchial “reactivity” at the core of the definition; that is the pathognomonic feature of asthma is excessive narrowing of airways due to contraction of ASM. It should be remembered that this was a decade before the advent of inhaled corticosteroids (6) and the majority of asthmatics at that time would have exhibited the characteristics of “poor control,” i.e., their lung function varies widely over short periods of time (6, 18). The adrenalin pressurized metered dose inhaler (pMDI) giving “*25% more FVC right now!”* as claimed in the adverts of the time^2^ (Figure 1) had been brought to the market just a few years before and this convenient, portable, and apparently safe medication, that could rapidly alleviate symptoms attributable to poor control, presumably influenced current thinking at the time. In this model the basic underlying abnormality lies in defective control of ASM tone and stimuli, such as an allergic response or viral infection triggering the excessive ASM shortening. This is analogous to the acquisition of a bacterial bronchitis in a well, cough free child with cystic fibrosis (CF)—the bacterial bronchitis does not define the condition but is both a consequence of the underlying condition and a driver of disease (symptoms and structural damage). The bacterial bronchitis with associated inflammation, if untreated, causes disease characterized by a chronic cough which may on occasions be accompanied by reduced energy levels leading to progressive damage of the airway wall that will eventually be manifest by the radiological appearance of bronchiectasis (which is not a disease but a radiological sign or pathological feature).

By the end of the 1970's the focus again moved away from the ASM such that many still referred to asthma as an “inflammatory” disease. This was largely attributable to the identification of IgE and its role in type 1 allergic reactions in the late 1960s and the demonstration by Dr. Harry Morrow-Brown in the early 1970's that an ICS (beclomethasone) could transform the quality of life of well-characterized “allergic” asthmatics (based on sputum eosinophilia) (6). For some time it was known that systemic steroids could have a dramatic effect on the disease, albeit with significant associated side effects, but the availability of an apparently safe, effective “anti-inflammatory” agent reinforced the notion that this almost magical effect was entirely mediated by its “anti-inflammatory” effects. By the early 1990's the British Thoracic Society guidelines (19) noted that “*Asthma is a common and chronic* ***inflammatory*** *condition of the airways. Its cause is not completely understood*. ***As a result*** *of inflammation, the airways are hyper responsive…”* As with subsequent GINA guidelines (20), inflammation was elevated to be the ***cause*** of asthma rather than a component and/or trigger factor.

Despite the intense investment of time, expertise and money this focus on asthma as an “inflammatory disease” has conspicuously failed to “solve” the origins and fundamental nature of asthma (though there is no shortage of hypotheses). This lack of clarity regarding the fundamental nature of the condition and the associated difficulties in agreeing upon a definition led to the British Thoracic Society guidelines abandoning all attempts at defining the condition and in essence defaulting to the position that asthma is something that gets better with asthma therapy^3^.

Does this matter? Unfortunately it does. The failure to define the condition leads to fashions, driven by “opinion leaders” keen to promote their latest “asthma paradigm,” which in turn contributes to the huge burden of over and under diagnosis experienced by patients (3–5, 21–23). Failure to apply clear diagnostic criteria means that findings of epidemiological studies are probably of little value and at worst can be very misleading (24). Much of the reported “asthma epidemic” of the late C20th appears to be attributable to diagnostic transfer. This is particularly true in the pre-school age group (24–26) with *wheezy bronchitis* (wheezing associated with a viral bronchitis—the wheeze being generated by airflow limitation as a result of accumulated airways secretions and to a degree, mucosal oedema rather than contraction of ASM) being mislabelled as asthma due to changes in diagnostic fashions (diagnostic transfer). As, with many unfortunate erroneous medical ideas of the past, this was driven by laudable intentions, which in this case, was an attempt to address the prevalent under diagnosis and treatment of asthma in school age children in the 1980s (26).

Attempts to hide our confusion has given rise to the suggestion that there are many forms of asthma with different “phenotypes” *[the observable properties of an organism that are produced by the interaction of the genotype and the environment]*. Pediatricians often have applied the term “phenotypes” to patterns of “asthma” and/or wheeze over time (27, 28), when in reality these “phenotypes” are not phenotypes at all, but merely retrospective arbitrary descriptions of temporal patterns of symptoms generally unrelated to underlying cause or other observable properties. Giving a condition a new name because we are having trouble understanding the underlying process is a game doctors have been playing for centuries.

If we had not identified the unifying, underlying defect in CF we would presumably have a similar growth industry with the various phenotypes such as typical CF characterized by malabsorption and lung disease, CF with diabetes, CF with liver disease etc. and we would be applying all kinds of powerful tools to study biopsies and pathways in and around the affected cells. At the same time we would have missed the diagnosis in the 15% or so who are pancreatic sufficient and indeed those turning up in adult infertility clinics who carry a mutation in the same gene.

## Why Should We Focus on the Instability of ASM?

*Understanding that loss of postnatal control of ASM homeostasis is the key component of asthma forms the cornerstone of accurate diagnosis and good clinical care*. The idea that a key component of “asthma” is excessive narrowing of airways due to contraction of ASM and that this can vary significantly over short periods of time goes back several centuries. ***It is the*** ***one feature that distinguishes the condition from all others***. Salter recognized this in 1859 (29), reflecting the consensus of those at the forefront of studying pulmonary disease at the time. He also recognized the importance of neurological control of the ASM tone.

## Components of the “Bronchial Hyperresponsiveness” that Characterizes Asthma

**Figure 2:**
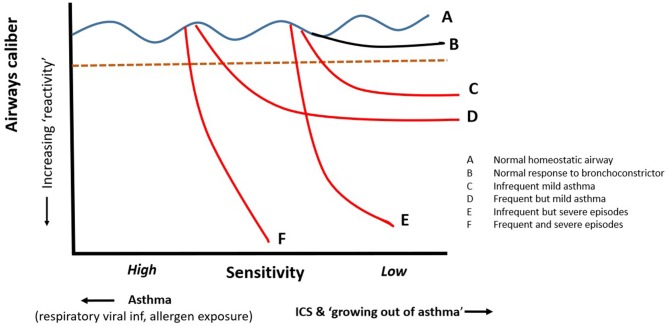
Bronchial hyper-responsiveness consists of two components; how *sensitive* the airways are to agonists driving shortening of ASM and how much the ASM shorten when the normal homeostatic mechanisms fail. The impact of shortening can be amplified in presence of significant airways secretions and plugging. The sensitivity and reactivity of vary between asthmatics and probably accounts for different patterns of symptoms from mild frequent to infrequent severe. Steroids have significant effects on sensitivity but may have little or no effect on reactivity once destabilization is triggered.

Asthmatics generally exhibit “*bronchial hyperresponsiveness” (BHR)*, which critically is composed of two components (Figure 2); increased bronchial ***sensitivity (BSen)*** characterized by a shift to the left on the dose response curve hence narrowing at lower levels of stimulation than non-asthmatic subjects' and increased bronchial ***reactivity (BRea)*** with asthmatic ASM shortening to a significantly greater extent than observed “healthy” subjects [(30–43); Figure 2]. “Bronchial responsiveness” (a term often used for BSens) varies in response to factors such as viral respiratory infections (39) and/or treatment with agents such as steroids (40–43). Indeed “sensitivity” appears to be increased following viral infections even in apparently healthy individuals (44, 45). In the absence of these factors bronchial “hyper-responsiveness” appears to be relatively stable over time (46, 47). Importantly, there does not appear to be a discrete, bimodal distribution separating asthmatics from healthy individuals (33, 34). While sensitivity appears to vary with viral infections and treatment with medication such as corticosteroids much less is known about whether bronchial reactivity changes in a similar manner not indeed whether it differs significantly between asthmatics and healthy individuals. Healthy individuals demonstrate a plateau effect when exposed to a bronchoconstriction stimulus which may be due to reaching maximal shortening but, more likely, this is due to homeostatic mechanisms preventing excessive shortening.

It is widely accepted that bronchodilator responsiveness as demonstrated by an increase in FEV1 of 12% or greater^4^^,^^5^ Main report: p19, will identify pediatric asthmatic subjects. The diagnosis can also be supported by a “positive” response to one of the numerous forms of challenge testing. The utility of placing ASM “hyperresponsiveness” at the core of the condition was clearly demonstrated in Finland where the introduction of a clear unified approach to diagnosis and management resulted in huge reductions in presentations to emergency departments (EDs), hospitalisations and apparently the virtual elimination of death due to asthma (48–50). They demonstrated that simple, consistent, and coherent adjustments to delivery of care transforms outcomes. In contrast, little or no progress has been made in countries such as the UK, USA, and Australia, which have devoted huge efforts to producing “guidelines but have not addressed failures in the delivery of healthcare.” Crucially, the Finnish “asthma programme” requires objective evidence that confirms the diagnosis before patients qualify for financial assistance with medication costs. Establishing a robust diagnosis with spirometry with bronchodilator responsiveness (or rarely a positive “challenge” test) is one of the central components of their enviable and very effective “asthma programme.”

The objective confirmation of ASM reactivity helped ensure that those with alternative diagnoses were not mis-diagnosed as asthma, thus denying them the appropriate intervention for their condition, whilst providing confidence in the diagnosis. Perhaps unsurprisingly there is evidence that a firm diagnosis is associated with improved adherence (51). It was also noted that the number of “milder” asthmatics increased suggesting that the programme also addressed, in part, the issue of under diagnosis (50). The one group in whom the “asthma” programme had little or no effect was in children under 5-years, in whom it is difficult to apply objective tests other than a dramatic and unequivocal response to asthma medication. Moreover, the number of “asthmatics” in early pre-school years represent only a small proportion of those who develop wheezing with a respiratory virus, with the majority having “wheezy bronchitis” (24, 52).

## “The Same Indefinite Application of the Term Still Remains in Vulgar Use” (10)

Depressingly, over- and under-diagnosis of asthma remains common (3–5, 21–23). The lack of focus on objectively documenting the presence of the key component of asthma is one of the principle reasons for over diagnosis. Conversely a failure to recognize that the degree of bronchial reactivity (and hence bronchoconstriction) follows a bell shaped distributed amongst asthmatics, with many having relatively mild constriction when triggered, results in under diagnosis at the milder end of the spectrum. Children with relatively mild asthma may not wheeze with intercurrent viral illnesses and present with coughing that takes many days or weeks to regress to the mean. Hence their symptoms are all too often dismissed as “just another viral infection.” Additionally, the ability of subjects to adjust to impaired lung function and thus minimize symptom reporting results in ongoing under-diagnosis. It is not rare to see an adolescent with an FEV1 of 54 and 40% reversibility who show no overt evidence of respiratory distress, are free of wheeze and who state they are fine and don't know what the fuss is about.

The lungs have a very limited repertoire of responses which contributes to the high prevalence of over diagnosis in many countries. Symptoms and signs commonly associated with asthma such as shortness of breath, chest tightness, coughing, and “wheezing” are also manifest in patients with a variety of other conditions. These include “wheezy bronchitis,” most common in the early preschool years (24, 52, 53); persistent bacterial bronchitis (PBB), again most common in the early years of life but occurring at all ages (54, 55); dysfunctional breathing common amongst competitive teenagers but also common in adolescents and adults with and without asthma; paroxysmal vocal cord dysfunction (pVCD) and related conditions such as exercise induced laryngomalacia (56–59); reaching ones physiological limit during exercise (58–60) (reported by individuals across the spectrum from unfit obese individuals through to elite athletes); airways structural problems, particularly in infants and young children, including malacic airways, vocal cord palsy, subglottic stenosis, and cardiac anomalies amongst others. Unfortunately prescribing an inhaler and applying the label “asthmatic” is only too easy, particularly if the clinician is not aware of conditions such as a PBB or dysfunctional breathing.

In those with “difficult asthma” mis-diagnosis is commonly identified. The insistence on objective evidence such as a positive bronchodilator response or significant constriction during a challenge before the label applied to a patient moves from possible or probable asthma to definite asthma would greatly reduce the very high levels of over diagnosis. De-diagnosing asthma is one of the principle roles of many tertiary care clinics.

In those with *objective* evidence of asthma, “difficult” asthma (at least in childhood) is almost always attributable to either

1. failure to deliver the ICS to the lungs (poor regimen and/or device compliance, i.e., they do not adhere >80% of the time or they have an ineffective inhaler technique (61–63) or
2. having asthma *and a co-morbidity* such as those outlined above with symptoms caused by the co-morbidity being managed with ever escalating doses of medication (64).

## Caveats to Scadding's Definition of Asthma

**Figure 3:**
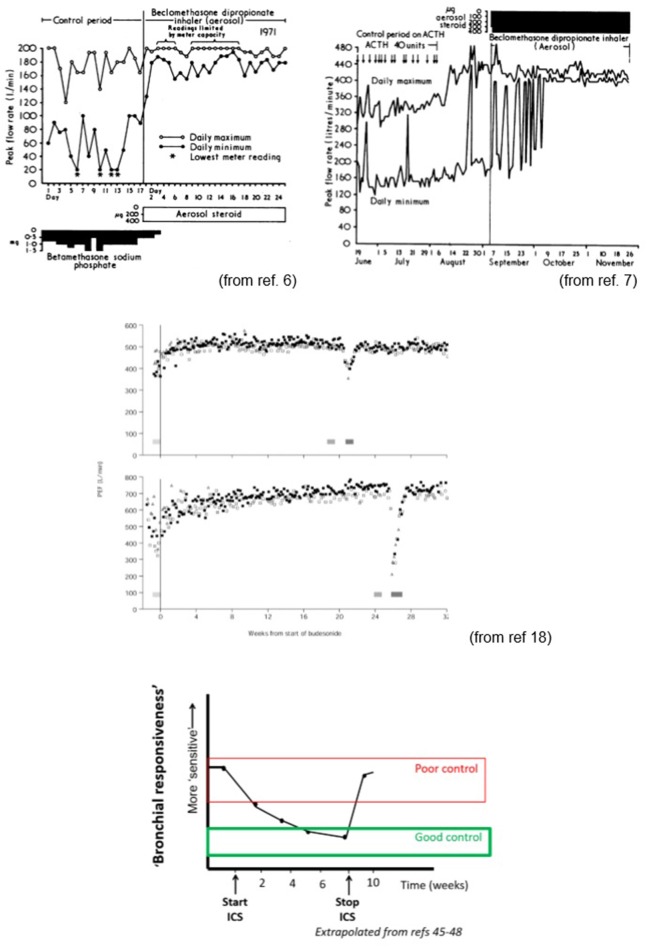
Impact of inhaled corticosteroids on lung function and bronchial responsiveness (sensitivity). Impact on sensitivity is relatively slow when commencing ICS but rapidly fades on discontinuing treatment. This largely explains the need for high levels of compliance (>80% of twice daily doses). Study by Reddell et al. (middle panels) indicates that even when asthma appears well controlled, ASM homeostasis can be severely disrupted during viral infections. The ability to induce/facilitate bronchoconstriction and the associated impaired bronchodilator responsiveness after the airways constrict might explain why the Finnish/Scandinavian approach of aggressive bronchodilator and ICS therapy early at first sign of increasing symptoms is successful at minimizing severe exacerbations and why combination products used intermittently appear to be helpful in mild to moderate asthmatics (65).

- Scadding and the American Thoracic Society (17) noted that asthma is a “*disease characterised by wide variation over short periods of time in resistance to flow in intrapulmonary airways.”* Wide variation in resistance to airflow *per se* is not sufficient, in that significant variations can be observed over relatively short periods in other conditions. For instance, a patient with moderately severe CF may experience a relatively rapid fall in FEV1 with an “infective” exacerbation. Conversely, poorly compliant adolescent CF patients with deteriorating lung function may have a rapid improvement in FEV1 over the first 24-h after admission with the institution of physiotherapy.
- Equally important is the failure of many clinicians to recognize that one of the key characteristics of asthma is that there is a fundamental difference between ***poor control*** and a viral induced ***exacerbation*** (or “spasmodic” asthma associated with catarrhal as Dr. Williams described it (1) or commonly referred to as an “attack”). This difference was highlighted in early papers reporting the effect of an inhaled steroid and was later highlighted by Reddel et al. (18) (see Figure 3). Patients with poor control often exhibit highly variable lung function changing rapidly over time reflected in diurnal peak flow measurements or rapid onset and relief from symptoms brought on by exercise (65) or allergen exposure. Generally, such patients are not on ICS or (more commonly these days) are poorly compliant.

In contrast during a “viral” “exacerbation” the infection of the airways appears to result in *both* a destabilization of bronchial ASM homeostasis resulting in bronchial ASM narrowing *and*, ***crucially****, a* tendency for those airways to become “locked down” such that they respond very poorly to β-agonist (18). Patients often describe an viral exacerbation of asthma as an “asthma attack” analogous to the use of the term “heart attack” to describe a myocardial infarct.

If the bronchodilator responsiveness were maintained patients would not require increased inhaled or systemic steroids even if the virus initiated bronchoconstriction. In those with the most severe “exacerbations,” heroic amounts of β-agonists at best produce small reductions in airways resistance (as well as significant side effects such as tremor tachycardia anxiety, hypokalaemia etc.). In a study assessing systemic salbutamol levels in patients dying of asthma in hospital it was demonstrated that lack of a β-agonists was not the cause of death, rather the patients died despite massive doses of β-agonists (66, 67). Of course the impact of viruses on the ASM function represents a spectrum from a mild but discernible increase in BHR in many “normal” individuals to the severe locked down life threatening attack experienced by a minority of asthmatics who have very significant reactivity. Other milder asthmatics do not experience such sever falls in FEV1 and often retain a degree β-agonist responsiveness.

In severe exacerbations systemic corticosteroids are required to “unlock” the ASM and restore the β-agonist responsiveness. The mechanism of action is unclear as corticosteroids have little impact on the neutrophilic inflammation typical of viral respiratory infections (52, 68) and have no intrinsic “anti-viral” effect. It should also be noted they appear to work equally well for both atopic and non-atopic asthmatics, suggesting that they are not working on “allergic inflammation.” Their impact on ASM can be detected within an hour, far too quickly to work through translational pathways. One group has suggested that the loss of bronchodilator responsiveness is due to loss of β-receptors resulting from viral RNA inducing prostaglandin synthesis via Toll receptors (69–71) (though these patients also fail to respond dramatically to anti-cholinergic drugs).

Inevitably, this is not an “all or nothing” effect. Clinical observation would suggest that asthmatics at the mildest end of the spectrum have mild increase in symptoms with viral respiratory infections and never experience a severe exacerbation. Others cope well with most viruses but have an occasional severe exacerbation and others have significant exacerbations with each respiratory virus. Along with this it will be observed that many, usually at the milder end of the spectrum, report reasonable if not compete relief when inhaling a β-agonist during a viral respiratory tract infection and others, generally those labeled severe, rarely deriving much benefit for their bronchodilators once an exacerbation has been established.

Primary care data suggest that perhaps only 14% of “asthmatics” have an exacerbation requiring oral steroids during a given year (at least in countries such as the UK) with the best predictor of a significant exacerbation being a previous such episode (72–74). The “severe” exacerbation presumably requires both significant reactivity (a tendency to large falls in airflow) together with a significant loss in bronchodilator responsiveness—small falls in airflow are unlikely to be life threatening and even large falls can be managed if bronchodilator responsiveness is largely preserved. The Finnish programme advocates aggressive use of β-agonists and ICSs as soon as a patient experiences increasing symptoms, an approach which they believe has made a major contribution to both the dramatic reduction of ED presentations and the very low use of oral steroids. It is reported that only 2% of asthmatics receive oral steroids in a year.

## Objective Confirmation of Asthma

In the absence of having identified the central component of asthma that leads to loss of ASM homeostasis and hence excess constriction, we are left with using physiological makers. We can measure blood glucose to diagnose diabetes, which provides a marker of disturbed glucose homeostasis resulting from an absence of insulin in type-1 diabetes or insulin resistance in type-2 diabetes. In type-1 diabetes we can measure insulin levels but in type-2 diabetes we need the physiological read out of elevated blood sugar levels. For those with CF the physiological read out of elevated sweat chloride (the “sweat test,” which replaced licking the infant) has long been the test of choice. However, with the recognition that CF is caused by mutations in a particular gene we have much greater insight into the spectrum of disease severity and manifestation.

The physiological read out for asthma is excessive lability of airways caliber attributable to loss of homeostatic control of ASM. Home peak flow measurements have been to help identify this lability though the high variability in peak flow amongst normal children makes this approach unreliable. As noted above more robust diagnosis can be based on an increase in FEV1 of >12% following inhaled short acting β-agonist Occasionally bronchial challenge testing is required to confirm bronchial hyper-responsiveness as for example, when high level athletes are seeking permission to use certain medications.

The recent NICE guidelines in the UK recognized the problem of over-diagnosis and the need for more objective criteria before confirming a presumptive diagnosis of asthma but curiously, they proposed the measurement of exhaled nitric oxide FeNO^6^, despite the lack of robust evidence that would support its role as a tool that reliably identifies asthma (75). FeNo appears to be a good marker of atopy (76, 77) but does not appear to be a marker of asthma or airways “hyper-reactivity.” Induced sputum eosinophil and FeNO levels do not appear to correlate with each other and neither correlates with BHR or lung function (78). FeNO has yet to find a role in the routine management of asthmatic patients (79, 80). The usefulness of the more time consuming process of evaluating eosinophils in induced sputum is also questionable, with no evidence of its utility in children (81, 82) and no impact on symptoms or lung function in adults, although a trend toward reduced exacerbations has been reported. One group tried to apply the NICE criteria to a cohort of children and found that the NICE criteria failed to identify the “asthmatic” children though it should be noted that the definition of asthma was based on a physician diagnosis and prescription of a short acting β-agonist, a notoriously inaccurate means of establishing the prevalence of the condition (83).

The reason NICE failed to learn from the successful Finnish national asthma programme, in which objective confirmation of diagnosis using lung function testing was the key component, is unclear. It is a curiosity that in most specialties, the fundamental tests supporting their practice are readily available. It is easy to obtain a full blood count, blood glucose, CXR, or ECG, yet health care systems are yet to embrace the need for widely available, high quality lung function testing (LFT). Indeed, for many, it is much easier to get a CT scan or upper GI endoscopy than high quality LFTs, which should be as readily available as a CXR. The fact that this is not the case and the majority of children with asthma in primary care are neither diagnoses or monitored using spirometry (3, 84) is another example of the failure of the respiratory world to advocate for their patients and should be source of embarrassment. The study from the Netherlands (3) noted that only 15% of school aged children with “asthma” had spirometry prior to the study. They went on to show that the majority of “asthmatics” did not have asthma. A very recent study from the U.K. (84) highlighted the improvement in outcomes when routine spirometry was introduced into the routine assessment of asthmatic children in primary care.

## “Allergic” Inflammation Is Neither Necessary nor Sufficient for Development of Asthma

“Atopic” inflammation does not appear necessary for the development of asthma, with estimates of the prevalence of atopy amongst asthmatic populations ranging from <5% to 94% across countries and regions (85–97). In Westernized countries atopy is generally reported in around 40–60% or more of asthmatics while in developing countries much lower percentages are reported. Many countries in South America report a high prevalence of asthma despite few of these individuals manifesting atopic markers such as skin prick test positivity. Several studies have shown that despite increasing atopy within populations accompanied by increases in eczema and allergic rhinitis, over recent years the prevalence of asthma has fallen or remained static. The disparate changes in the prevalence of atopy, asthma and related “atopic” conditions again questions the central role of atopy in the generation of asthma (85–96). The explicit recognition of atopic and non-atopic asthma (97) reinforces the concept, that while atopy may be an exacerbating co-morbidity, it is not central to the manifestation of asthma. Association does not imply causality. One possible confounder is the weakness of questionnaires that rely on reported wheeze to help estimate the incidence/prevalence of asthma. To ask whether your child has had a wheeze or whistling sound in the past year is to invite a positive response if the child has had any noisy breathing even if it is not a “whistling” sound (98). As a result, the prevalence of asthma is probably greatly over-estimated (24) making assessment of trends over time almost meaningless.

Conversely, allergic inflammation does not appear sufficient for the development of asthma, in that the majority of those with evidence of “atopy” do not manifest the bronchial hyper-responsiveness central to manifesting asthma. Indeed the majority of those with an allergic rhinitis or eczema do not manifest asthma. It appears very likely that many individuals with a seasonal rhinitis due to grass pollen or perennial rhinitis due to an allergen such as house dust mite will have airways inflammation due to an *allergic bronchitis*. In the absence of significant bronchoconstriction these individuals may experience cough and some increase in airways secretions. Examination of the airways will reveal inflammation similar to that reported in “allergic asthmatics.” These individuals have an “allergic” or “eosinophilic” bronchitis but the stability of the airways is not significantly compromised and they do not bronchoconstrict (99–102). Some refer to these patients as having “cough variant asthma”. These subjects with an allergic bronchitis will respond to ICSs if their cough is troublesome (99–102). Despite corticosteroid responsiveness and some asthma *like* symptoms they do ***not*** have asthma in that they do not exhibit significant bronchoconstriction due to ASM activity.

It is also increasingly recognized that eosinophilic bronchitis is absent in many asthmatics (103–106) arguing against the eosinophil having an essential role in the development of asthma (thought they may well-contribute to symptoms). Conversely, in those who out-grow their asthma there is evidence that the inflammatory profile persists in most subjects studied suggesting that the AHR has altered and homeostasis has been restored despite persistence of ongoing inflammation (107–112). A study in asymptomatic subjects previously diagnosed with asthma found evidence that FeNO fell after introduction of inhaled steroids but, importantly, there was no change in clinical status (111), again questioning the link between FeNO and asthma *per se*. While several studies suggested this ongoing inflammation may predispose to relapse, there is no evidence to support or refute this suggestion. However, the discrepancy between re-established airways homeostasis and ongoing inflammation suggests the inflammation *per se* is not the critical factor.

The central role of the eosinophil has also been questioned through observations that early studies with anti-IL-5 agents in asthmatic subjects significantly reduced eosinophilia but had little or no clinical impact (113, 114). Eosinophilic asthma is variously defined as having >2 or 3% eosinophils in a BAL or induced sputum. Even in this minority of asthmatic patients there is debate as to whether driving therapeutic decisions on the basis of eosinophil numbers is any better than making changes on the basis of symptoms. More recent studies with anti-IL-5 agents have been shown to have an impact on the rate of exacerbations in highly selective populations of “severe” “steroid resistant” eosinophilic asthmatics but they certainly do not “cure” asthma and indeed appear to have very little effect on quality of life or lung function (113, 114).

Importantly there is no data to suggest that atopic status significantly impacts on the response to inhaled corticosteroids. The most effective therapy for those with asthma irrespective of atopic status are ICS suggesting that their action is not fundamentally through suppression of “atopic” inflammation.

In order to address the problem that atopy does not seem to be either necessary or sufficient for the development of asthma, those committed to the idea that “TH2 inflammation” causes asthma have proposed that in those without atopy the inflammation underlying the condition is commonly driven by type 2 innate lymphoid cells (ILC2s) that generate TH2 type cytokines (115, 116). However, the same considerations relating to the paradigm that asthma is a TH2 “allergic” disease pertain to the role of ILC2 cells. Moreover, should they prove to have a role, it is unclear why some individuals should develop ASM hyper-responsiveness as a consequence of the activity of this type of cells when they are present in all individuals.

Given that inflammation *per se* does not define asthma, those who believe atopic inflammation or certain infections in early life or dysbiosis of gut and/or lung microbiome causes asthma (rather than being a exacerbating factors) need to move from studying inflammatory/immunological pathways and concentrate on how they might disrupt the normally robust ASM homeostasis.

### “Non-atopic” Asthma in Pre-term and Small for Gestational Age Infants

Pre-term individuals are an interesting group, in whom there is a significantly higher rate of asthma (as defined by demonstrable bronchodilator responsiveness) than the general population and in whom atopy appears not to be a significant risk factor (117–121). Around 20% of such individuals may demonstrate significant bronchodilator responsiveness. There is evidence of on-going neutrophilic (but not eosinophilic) inflammation in the majority of these patients (117, 122–127) well into childhood, but the relevance of this, if any, to the development of asthma in a minority, is unclear. In one study reporting increased sensitivity to exercise, the fall in FEV1 in those born preterm was <7% in the vast majority, which may be related to structural changes and effects of gas trapping rather than bronchoconstriction (126). Interestingly, the impact on airways responsiveness appears to be greatest in those born small for gestational age and not related to “BPD” status (125).

Asthma in those born pre-term has not been studied in detail and while inhaled steroids and ICS+ LABA are widely used (127) there is no published evidence to support their use in this group. Older patients often report no significant benefit when started on ICSs which may imply that in many the BHR may not improve with steroid or that years of adaptation to low lung function has resulted in physiological adaptation as with severe poorly controlled asthmatic or may reflect the impact of the CLD structural changes as a co-morbidity. There are a number of on-going studies addressing therapeutic approaches which may provide valuable information.

## Loss of Effective Airways Smooth Muscle Homoeostasis—The Key to Understanding Asthma

As will be argued in more detail below, the fundamental defect leading to asthma appears to be a loss of the normal post-natal homeostatic control. Antenatally, ASM contracts vigorously and frequently with regular peristaltic waves moving distally from even in the earliest stages of lung development. This is vital for lung development but toward term these peristaltic waves cease. In contrast to it its marked physical contractions prenatally, post-natal ASM maintains a relatively constant length with relatively minor and non-coordinated oscillations around an optimal length, probably controlled by classic negative feedback loops. To date no clear role for ASM in a healthy individual after birth has been identified. The most likely role, if any, is likely to be helping to maintain an optimal luminal diameter in the conducting airways in order to minimize resistance to airflow while at the same time minimizing the dead space within the conducting airways.

### Airways Smooth Muscles Cells Are Not a Unidimensional

Airways smooth muscle cells are generally considered to be as highly specialized cell whose function is essentially confined to shortening or lengthening with little or no other contribution to the function of the lungs. However, there is a robust body of work suggesting that ASM have other functions and capabilities including the ability to produce a range of cytokines, including IL-13, IL-5, and IL-8 and a range of proliferative cytokines in respond to a variety stimuli such IL-1, IL-13, TNFα, IgE mediated activation and LTB4 (128–144). Hence it would not be a surprise that an inflammatory response could be generated by unstable or activated ASM which may be independent of/or additive to other inflammatory processes such as an atopic bronchitis. Moreover, bronchoconstriction and the resultant stress on cells such as epithelial cells and fibroblasts may also result in release of inflammatory cytokines secondary to the constriction.

Cytokine release by ASM and/or associated stressed cells may be the key link between excessive constriction secondary to loss of homeostatic control and the evidence of inflammatory response in the “asthmatic airway” and would explain the inflammation frequently observed in “non-atopic” individuals with poorly controlled asthma. The “airways inflammation” may of course be augmented by “allergic” responses to allergens or neutrophilic responses to viral and/or bacterial infections (PBB or perturbation of the “healthy microbiome” occurring in some poorly controlled asthmatics presumably due to the associated impaired mucociliary clearance and plugging) (54, 55).

A super-added inflammatory processes such as an “allergic bronchitis” may, in those in whom homeostatic control of airways caliber is impaired, contribute to symptoms by driving constriction through the release of mediators such as histamine and cysteinyl leukotrienes (cLT). It is also possible, but not proven, that this pattern of inflammation can, in *some* individuals, contribute to the development of loss of homeostatic control of ASM. Despite the enormous financial and intellectual investment in trying to “prove” the postulated link between an “allergic” Th2 type inflammatory response and the *causation* of asthma (as opposed to inducing symptoms) direct mechanism linking the two have not been established.

While the natural assumption is that local mediators predominantly drive the constriction observed during “allergic asthmatic reactions” highly selective vagotomy appears to prevent bronchoconstriction and asthma in a dog model of allergic asthma (145) while a significant proportion of the constriction due to methacholine appears to be mediated by a parasympathetic reflex activity that maybe important in humans (146).

Asthma does not appear to be analogous to type-1 diabetes, in which a key component of normal homeostasis is permanently lost. Rather it seems more similar to the development of type-2 diabetes, which may or may not be evident in individuals of the same BMI. The same relative obesity does not lead to the manifestation of a condition in all individuals with a number of factors influencing whether homeostasis is maintained. In subject of the same sex age and BMI some will have no identifiable problem with glucose homeostasis, others only when stressed and some will have very poor glycaemic control. Similarly removal of stimuli can result in greatly improved homeostasis (improved diet or allergen avoidance, respectively), while control can be fully restored by life style changes in case of type-2 diabetes and by taking ICSs or growing out of asthma through mechanisms unknown. As already noted, ASM has been shown to respond *directly* to corticosteroids with reduced cytokine production and changes in bronchial responsiveness, raising the possibility that a major effect of ICSs is on ASM (147–153). This is potentially most evident during exacerbation when they can positively influence ASM responses to bronchodilators when the ASM is exposed to viral RNA in the absence of any other cell type. The onset of discernible effects in asthmatics occurring within minutes would again suggest that there may direct effects on ASM cells, since the transcriptional changes influencing the “allergic inflammation” would be expected to take much longer (though clinically significant changes take longer).

### What Is the Role of ASM, If Any, in Healthy Airways?

Some have argued that ASM is simply a vestigial tissue with no significant function in highly evolved mammals (154, 155). This argument was one of a number used to promote the concept of thermoplasty and muscle destruction as a potentially safe approach to the treatment of asthma (156–158).

Smooth muscle is distributed extensively in the primitive lungs of lung-fish and amphibians as well as the swim bladders of fish. In the earliest air-breathing fish and amphibians, the primitive lung's airways smooth muscle contributed significantly to emptying the lung sacs. Air is actively forced into the primitive lungs by muscles in the upper airways while emptying is also an active process driven by largely the smooth muscle of the lower airway (159–164). Of note, much of the control mechanisms via the vagus nerve appears to have been established at the very earliest stages in the evolution of the lung and air breathing, while surfactants also date back to the earliest lungs and swim bladders (165–171). The evolution of the diaphragm, present only in mammals, and a separate relatively rigid thoracic cavity, together with robust elastic recoil provided for a much more efficient means of filling and emptying the lungs (dinosaurs, birds, and reptiles developed completely different mechanisms for filling and emptying their lungs). Consequently, the need for active contraction of muscle intimately associated with the lung in order to exhale air was negated. In diving mammals, ASM appears to act with cartilage to stabilize the airways while air is forced out of alveoli into more central and upper airways structures due to the increase external pressure at depth. Emptying alveoli helps to both protect these fragile structures from barotrauma and prevent nitrogen narcosis. Surfactants appear to help unstick the closed alveoli, a role they probably served in the earliest rudimentary lungs (165, 166).

This led some to suggest that persistence of ASM may represent an evolutionary curiosity with no contemporary function but retaining the potential to cause harm analogous to the appendix (154, 155). However, antenatal ASM plays a critical role in lung development grounded in millennia of evolution (154–163) and post-natally it probably plays a central role in the highly efficient process of inhalation and exhalation through maintaining an optimal configuration of the airways.

### Function of ASM *in utero*

Airways smooth muscle appears very early in fetal development and exhibits spontaneous rhythmic contraction and relaxation as well as having functional cholinergic innervation (172–191). Of note, *in utero*, the peristalsis is proximal (larynx) to distal, as is the case in the gastrointestinal (GI) tract from which the lungs develop. The pressure generated at the tip of the lung buds by this peristalsis acting on intraluminal fluid appears critical to lung growth and its absence results in hypoplastic architecture. Indeed complete inhibition of ASM prenatally is not compatible with life due to severe lung hypoplasia. The branching structure of the conducting airways is complete before term with post-natal growth being largely in relation to the size of conducting airways and number and size of alveoli and associated respiratory structures (192). *In utero*, a proximal pacemaker appears to be operating, co-ordinating peristalsis in a distal direction (181, 182). There also appears to be differential control of new and relatively newly formed ASM and that in more established central airways. As the fetus approaches term the phasic activity progressively declines starting in the more central airways and essentially disappearing even in the most distal airway by term.

In primitive amphibians, SM played a role in both developing the primitive lung and emptying the lung. In mammals, the evolution of the thoracic cage with a powerful diaphragm to drive inhalation and use of elastic recoil of the chest to exhale fundamentally changed the means of inflating and deflating the lungs. Consequently, in contrast to the gut smooth muscle, ASM normally ceases to constrict in a coordinated manner shortly before term. This suggest that a fundamental change in control has occurred in which homeokinetic control is established in order to maintain a stable airway that minimizes the work of breathing air through minimizing resistance to airflow, while minimizing the anatomical dead-space of the conducting airways. An example of the post-natal resistance to significant narrowing (presumably through negative feedback mechanisms) is the very small change in airways resistance noted in most normal individuals even when inhaling very high doses of agonists such as methacholine. One would predict that establishing stability of mammalian airways in post-natal life would be the norm, as there would not appear to be any evolutionary advantage in having the airways contract sufficiently to impair potentially life-saving activities such as running away from a predator!

### Post-natal Function

Birth brings about major changes with the fetus transitioning from a submerged life to an air breathing terrestrial existence. The surges in catecholamines associated with birth are one of factors driving the physiological and functional changes. Fluid is rapidly removed from the airways, oxygen is obtained from the lungs and there are major changes in vascular resistance and circulation. The epithelium of the conducting airways undergoes significant changes from the relatively primitive squamous epithelium to a complex post-natal epithelium and it is very likely that their functional activity changes—changes probably paralleled when taking submerged bronchial epithelial cell cultures and exposing them to an air-liquid interface.

Post-natally, airways caliber is tightly controlled. There is evidence of relatively slow phasic contractions (164, 193–204), presumably due to ASM length oscillating around an optimal mean. These oscillations appear to be relatively small with little or no impact on total airflow resistance. In order to maintain an optimal airways caliber the ASM is presumably under some form of classic negative feedback control. There is little or no evidence that the acute lower respiratory illnesses so common in the first year of life evoke significant bronchoconstriction further strengthening the suggestion that asthma is an acquired dysfunction of ASM. This is despite clear evidence that infants have functional ASM which respond to agents such as histamine and effective β-agonist responses which can block constriction in response to these agents (205, 206). Indeed infants, soon after birth, may have relatively greater ASM in their airways than older subjects based on comparative animal studies (192).

It is unclear whether there is co-ordinated contraction and relaxation of ASM around or whether units operate independently. One possibility is that stability is achieved by ASM cells contracting randomly and independently with the lack of coordinated constriction producing stability of the airways (193). Of note, selective vagotomy abolishes both the baseline tone and the oscillation, indicating that centrally derived cholinergic innervation plays an important role in one arm of the homeostatic control of airways caliber.

While the lungs have evolved over millennia to operate as efficiently as possible it is argued that this optimal design places the airways at risk of significant functional limitation if the normal geometry is perturbed (207). The conducting airways take up as little space as possible and the configuration represents the least space consistent with minimizing resistance. However, relatively small decreases in caliber can have significant impact due to the resistance being related to the fourth power of the radius. This vulnerability will vary from individual to individual based on their airway morphology. This potentially contributes to differences in the impact of similar degrees of airways shortening on lung function in different individuals. One mechanism that may be important in limiting the potential harm that can arise from excessive constriction lies in the partial helical orientation of ASM which results in shortening as well as narrowing of the bronchi which appears to limit transverse narrowing (208).

“Deep breaths” can “reset” the system and reduce the propensity to bronchoconstriction (30, 209, 210) though the effect is less in asthmatics and indeed in more severe asthmatics frequently induces bronchoconstriction (211, 212). This may be mediated centrally with anticholinergic therapy being reported to abolish the increase in BHR observed in asthmatics following breath holding.

The transition from the submerged environment in utero to air breathing independent existence parallels, in many aspects, the evolutionary transition from a wholly aquatic existence to land dwelling creatures. Understanding how this profound developmental change is established, perhaps through comparing pre-natal and post-natal function; epigenetics or gene expression, may finally give us the answer to what is the fundamental defect underlying the development of asthma in post-natal life.

## Factors Influencing ASM Stability

Amongst those with asthma the loss of homeostatic control can be largely restored by treatment with corticosteroids while for many children and adolescents with asthma the excess ASM constriction can resolve spontaneously and are often said to have “grown out” of their asthma (213, 214). This would suggest that cure might be possible if we genuinely understood the homeostatic mechanisms in healthy airways. In some of these individuals, asthmatic symptoms will reoccur at a later date. As has been recognized for centuries there is a hereditary predisposition to the disease, but environmental factors are clearly essential for initiating and perpetuating the condition. While there is intense interest in perinatal/early life effects asthma can develop at any age, with a very large proportion (probably the majority) of adult asthmatics apparently developing the condition after childhood (19, 215, 216). The onset of occupational asthma in previously well-adult individuals^3^ further emphasizes the potential for loss of homeostasis at any age.

It is of note that the key benefit derived from regular, effective ICS therapy is to re-establish normal ASM homeostasis—the significant variations in airways caliber over short periods resolving over a number of weeks with ASM “hyperresponsiveness” also being restored to or near “normal” values (6, 18, 40–43, 217–227). As part of this some appear to re-establish a “plateau” in the dose response curve when inhaling an agent such as methacholine (222) though the greatest effect is on sensitivity. This results in less day to day “bother” (diurnal night-time symptoms, exercise induced symptoms etc.) and at least partial protection from exacerbations (72, 220, 221). To achieve this it would appear that patients with significant symptoms need to take at least 80% of doses (that is at least 11 of 14 doses a week for a twice daily regime) (223) something that is achieved by <80% of the population in most studies. Below this figure the treatment effects are often completely lost. It is an indictment of our lack of progress that we still do not know whether 100% adherence would eliminate symptoms and prevent all significant exacerbation in the vast majority of asthmatics subjects.

Studies indicate that lung function variability improves more quickly than BHR [(40–42); Figure 2]. The greatest impact on lung function is on the daily trough (Figure 3). The large falls from one's best being eliminated in those with good control. In many the maximum flows also increase after introduction of ICS, this effect being seen in most in those with the more troublesome asthma. Much, but not all, of the benefit from commencing ICS, in terms of reduced BHR, is achieved within the first 4–8 weeks. This is somewhat slower than would be expected should the effects be attributable in large part to their “anti-inflammatory” effects again suggesting a disconnect between “inflammation” and BHR. Evidence suggests that BHR may continue to improve over a year and if ICS are stopped at this point lung function and BHR may return to previous values though this may be a little slower than after a shorter course (218, 224). In contrast to the relatively slow onset of action, the effect of ICS in restoring homeostasis and preventing the day to day variation in caliber attributable to ASM activity is, intriguingly lost within days of discontinuing them after short term treatment.

Critically, these agents appear to work just as well in non-atopic “asthmatics” as in atopic subjects which again strongly suggests that they have a direct impact on ASM function independent of any “anti-inflammatory effect”.

### Is Loss of Homeostasis and Hence Development of Asthma Reflected in Bronchial Sensitivity and the Magnitude of Airflow Obstruction, and Hence “Severity”, a Function of the “Reactivity”?

#### Bronchial Sensitivity [*BSen*]

The observations that asthma can develop at any age and that many children “outgrow” their asthma, again suggests that “sensitivity” is not an absolute value and that it can be influenced by environmental factors, possibly via interactions with the host immune system and/ or epigenetic changes. Bronchial sensitivity appears to have two components; inherent baseline sensitivity and a more labile variable component. As with all physical and physiological traits there is likely to be a normal innate distribution of inherent sensitivity to constrictor stimuli. However, there is ample evidence that an individual's bronchial “sensitivity” can vary over short periods of time, exemplified by the changes in sensitivity following the commencement and cessation of steroid therapy or the development of an inter-current respiratory viral illness. As noted above, this can affect “bronchial sensitivity” even in healthy individuals, though the effects are generally not marked. In those with an “allergy,” it can also be observed following a laboratory allergen challenge or during seasonal exposure to an allergen such as grass pollen. Conversely allergen avoidance such as moving to high altitude for house dust mite sensitive individuals appears to be associated with a reduction in sensitivity (228).

The speed of onset and offset observed in carefully controlled studies of ICS use suggests they do not simply act through their “anti-inflammatory” actions but may have direct effects of ASM and their “sensitivity.” In a detailed double-blind placebo controlled study (41), an improvement in PC20 of 1-doubling dose was noted within 6-h of the first dose of inhaled corticosteroid [ICS]. This was accompanied by a 0.2 L increase in FEV1. In a similar study a significant improvement in PC20 was observed at 12-h when compared with placebo (219). By 42-days the PC20 had increased by mean of 3.4-fold (with a 0.53 L increase in FEV1). Within a week of discontinuing therapy, the PC20 had returned to placebo values (40). Much of the improvement in FEV1 occurred in the first week while BHR was still improving significantly at 42-days—long after the “anti-inflammatory” effects of ICSs should have had their effect. Indeed, in long term studies the improvement in BHR is most marked during the first 4–8 weeks but it continues to improve, albeit much more slowly, over many months. Short term studies suggest the improvement in “BHR” are lost over a matter of days following cessation of the ICS (41, 219). For those who have been prescribed ICS for a number of years the rate of reversion to the mean appears to be slower but still occurs.

Evidence that the underlying B*Sen* can be altered for prolonged periods or indeed permanently comes from work in the field of occupational asthma with subjects who develop symptoms and who are then removed promptly from the precipitating allergen may lose both symptoms and “BHR,” while those exposed for a period of time appear to exhibit both persistent asthmatic symptoms and “BHR” despite allergen avoidance and ICS use (228–230). The increased BHR [sensitivity] and asthmatic symptoms can persist even if the specific IgE and bronchial response to challenge with a particular agent resolves. The resolution of the acquired increase in B*Sen* with early removal of the occupational allergen and the increasing likelihood of permanent changes with more prolonged exposure suggests that a component of the persistent/recurrent exposure induces significant changes that result in a persistent alteration in the B*Sen*. This observation does not however determine whether there has been a permanent change in ASM sensitivity *per se* or whether the effect is mediated through other changes in the host, such as ongoing inflammation in the absence of the initial driver.

#### Bronchial Reactivity [B*Rea*]

In comparison with B*Sen* far less work has been undertaken regarding the nature of reactivity and how it is influenced by factors such as inhaled corticosteroids and allergen exposure. Importantly we do not know whether changes in B*Sen* are paralleled in changes in B*Rea*.

It appears that the “normal” human airway has the ability to respond to non-specific stimuli such as histamine but individuals rapidly reach a plateau beyond which further constriction does not occur. Any reduction in lung function is generally minimal. Very high doses of histamine, for example, can induce systemic side effects such as flushing and marked tachycardia in the absence of further constriction suggesting the driver to constriction is substantial but the normally homeostatic mechanism are very effective at resisting bronchoconstriction.

Amongst asthmatic subjects there appears to be a spectrum of responsiveness with many exhibiting a plateau in their maximal constriction such that while symptomatic further doses do not lead to death (Figure 2). The concept that an individual's *BRea* in large part determines the severity (rather than frequency) of symptoms would be consistent with the observation that the best guide to future significant exacerbations is previous exacerbations—those who have had severe episodes before are likely to have them again. While this may reflect factors such as adherence to therapy it is also likely to reflect the reactivity of the airways, the impact of a virus being much greater in someone whose airways are able to constrict sufficiently to reduce FEV1 by 70% than in someone whose maximal fall is 18%. What determines “reactivity” is unclear but is likely to be related to muscle mass and other components of the complex pulmonary structures.

Some have suggested that the “plateau” is an illusion that reflects a failure to deliver sufficient concentration to the mucosal surface with nebulised therapy. In support of this, Brown et al. demonstrated, in an *anim*al model, complete local occlusion of a bronchus with local topical application suggesting that all airways can be closed even those with significant quantities of cartilage (231, 232). It should be noted the closure was limited to the area of application. In support of this suggestion is the observation that a significant number of asthmatic deaths and near death episodes appear to be acute asphyxiating episodes often following exposure to a particular allergen. An individual who is cyanosed and unconscious may be walking and free of significant respiratory distress a short time after an injection of adrenalin, suggesting the homeostatic mechanisms are overwhelmed but the ASM is not locked down, as occurs in those with viral exacerbations.

Although ICSs influence “bronchial responsiveness” primarily through changing sensitivity to agonists, there is relatively little information regarding their impact on reactivity and maximal changes in airway geometry once the homeostatic mechanisms are overcome. While ICS therapy does tend to increase the maximum PEF, it has a much greater impact on the minimum PEF, largely abolishing the significant diurnal variation characteristic of many with asthma (Figure 3). When the ICS are discontinued the minimum morning PEF generally returns to approximately pre-treatment levels suggesting that there is no fundamental change in reactivity.

Similarly, it is noteworthy in the examples shown in the Reddell study (18) that during a period of apparent “good control” the minimum PEF observed during acute exacerbations were similar to that observed during the period of poor control. For one subject this equated to a severe life threatening viral induced exacerbation, but prior to introduction of ICS a similar degree of airways obstruction as assessed by PEF was merely a nuisance rapidly responding to an inhaled β-agonist. This suggests that once the tipping point required to destabilize the ASM is reached, it will constrict to a similar degree to that seen in periods of poor control. Airflow limitation that is a nuisance for the poorly controlled asthmatic, becomes severe and potentially life threatening due to the loss of β-agonist responsiveness with the amplifying effect of secretions accumulating in the airway.

## Implications for Managing Exacerbations and Preventing Deaths

Asthmatics deaths have been categorized into short or long interval death, that is death within 2 h of onset of significant symptoms and those in whom symptoms have been present for longer, usually >5–8 h. There are clearly confounders such as failure to perceive deterioration in poorly controlled subjects. Despite these, post-mortem studies suggest that there are differences, with short duration deaths being characterized by greater mast cell degranulation and neutrophils with fewer eosinophils and less mucus (233–236). As noted above, this suggests that many rapid deaths are predominantly due to acute constriction, which may or may not occur on top of signs of poor control. In those who die after a longer duration, difficult to clear mucus accumulates in narrowed, severely inflamed airways that have lost their β-agonist responsiveness and in which mucociliary clearance is grossly impaired with patients effectively drowning in secretions.

One clear finding from the Finnish experience^4^^,^^5^ (49, 237) is that escalating therapy, usually with increased ICS doses as well as β-agonists, at the first sign of increased symptoms at the onset of an exacerbation, usually prevents progression to significant constriction and severe symptoms. The Finnish action plans are far more robust than those promulgated in other countries, with patients advised to take action at the first sign of deteriorating symptoms and/or a viral infection. Not only does this reduce presentations to ED, it said to have virtually eliminate asthma deaths and greatly reduces the use of oral steroids for exacerbations [2–4% of asthmatics receiving oral steroids in a year (49, 237) compared with 14% or more in the U.K]. This suggests that being aggressive before lung function starts to fall significantly with a viral exacerbation is effective in stabilizing the airways, through combining bronchodilation and increased steroid exposure. Combination ICS/LABA inhalers are generally more effective than high dose ICS alone in preventing significant exacerbation and this may be due to the dual action of both inhibiting constriction and helping to maintaining responsiveness. Two very recent, large randomized studies in adults and adolescents with mild to moderate asthma have found that using combination therapy with a rapid onset β-agonist with symptoms was as good or better than maintenance therapy with a short acting b-agonist for increased symptoms (238, 239) This may be due to the LABA helping to maintain airways caliber with the high dose ICS helping to maintain β-agonist responsiveness thus preventing the potentially large and difficult to treat falls in lung function that characterize severe exacerbation. Given the very low levels of adherence to maintenance therapy this is probably is what is happening in real life and is likely to have driven the popularity of combination ICS/LABA therapy in primary care.

## Does ASM Mass (With or Without Hyperplasia or Hypertrophy) Play a Role in Severity?

Assessment of ASM mass in post mortem specimens, resected lungs, and imaging suggests that asthmatics appear to have more smooth muscle than non-asthmatics (67, 240–249). However, as with BHR there is no evidence of a clear bimodal distribution in ASM mass, with values overlapping with the normal population and hence ASM mass alone will not “cause” asthma. Rather relatively greater ASM mass may, with other factors such as airways size, influence the magnitude of narrowing if the homeostatic state is destabilized. It may also explain in part the differences in severity of airflow obstruction noted across the spectrum of asthma severity.

It is likely that, as with other biological variables, the amount of ASM is normally distributed amongst healthy individuals. One prospective study suggested that ASM mass in early childhood is the best predictor of asthma later in childhood (250), suggesting that ASM mass maybe a risk factor for the manifestation of asthma, though this was not replicated in a second study (251). The limited available data from adults suggests that the ASM bulk in asthmatics is related to severity and not duration of asthma (244). If this is the case, it suggests that the more severe asthmatics have a pre-existing relative increase in ASM though it is also possible there may be an early increase in muscle mass but the ability to increase in size is limited and hence not progressive. If there is an increase in ASM mass, is this due to hyperplasia rather than hypertrophy? Some studies have suggested that ASM cells increase in number, but to date there has not been any evidence of increased proliferation *in situ* (252). For skeletal and cardiac muscle, the response to increased load appears to be hypertrophy rather than hyperplasia.

Animal experiments do not suggest significant changes in muscle mass, lung function, or ability to change the force generated with repeated ASM constriction, even though other “remodeling” changes such as increases in goblet cells and “basement membrane” are observed (253, 254). These findings are similar to observations in a short-term human study (255), though it should be noted that even after the repeated stimuli, the values in those who had repeated constriction were similar to those of the controls. Of note, they did not assess the effect on ASM. A subsequent study involving repeated bronchoconstriction in adult asthmatics was unable to identify any impact on lung function (256).

In studies reviewing the impact of thermoplasty (257, 258) it is clear that ASM mass in larger airways is reduced post procedure, though it appears that the improvement in control does not correlate with the reduction of mass (258) and hence maybe in part due to other effects including those on ASM innervation. The greatest impact appears to be on the frequency of events associated with significant contraction (reflecting reactivity) such as reduced oral steroids use, ED presentation and hospitalization. Pre and post bronchodilator lung function was largely unchanged (257). One study, which looked at “equine asthma,” found that allergen avoidance and inhaled steroids did lead to a small reduction in ASM (259). Interestingly, they found avoidance was better at dealing with the inflammatory component, while ICS had a greater impact on the ASM mass, again suggesting a degree of disconnect between inflammation and ASM activity and that ICS may well act directly on ASM.

ASM may be altered in other ways; resting tone and length may be re-set or its function may be altered with more rapid recycling of contractile units producing a greater effect for a given stimulus. Evidence suggests that ASM show a significant degree of plasticity and do not have a force-response curve typical of skeletal muscle. Asthmatic ASM does not appear to generate greater forces for a given stimulus than that from non-asthmatics (260, 261). Krishnan et al. suggested that asthma represents “freezing” of smooth muscle (262), but “freezing” only appears to occur during viral infections when airways obstruction is relatively fixed. During poorly controlled asthma, the variability in caliber is greatly enhanced by specific and non-specific constrictor stimuli, quite the reverse of being “frozen.”

## Autonomic Innervation of the Airways

It is well-established that in the GI tract there is parasympathetic innervation which exerts both excitatory and inhibitory control on the tone of the smooth muscle as well as sympathetic innervation that has a predominantly inhibitory effect during extra-uterine life (263). However, in the case of the human respiratory tract, sympathetic innervation is sparse and predominantly supplies blood vessels and submucosal glands. In contrast to humans, spinal adrenergic sympathetic nerves supply ASM in some mammals such as guinea pigs, cats and dogs (165, 264, 265). In humans it is the cranial parasympathetic nervous system that predominantly controls the ASM through extensive plexuses. The vagus nerve carries both cholinergic and non-adrenergic non-cholinergic (NANC) nerves, activation of the former leading to constriction and the later to relaxation. Selective vagotomy results in loss of the normal rhythmic contraction of ASM and bronchodilation. Given the position of parasympathetic ganglia which are predominantly located along the trachea and large bronchi, it is possible the effect of thermoplasty may be on neuronal control of ASM.

Nitric oxide [NO] and vasoactive intestinal peptide (VIP) act as the principle transmitters in the mammalian NANC parasympathetic bronchodilating system with the former predominating in humans (though to date there is no direct evidence that NO gas is released during these responses). It appears that parasympathetic mediated relaxations of ASM requires higher frequency of stimulation than those needed to evoke parasympathetic cholinergic contractions (165, 264, 265).

The lack of spinal adrenergic innervation capable of contributing to control of ASM tone may place humans at particular risk of loss of homeostatic control particular given the potent potential constricting effects of cholinergic innervation. Impaired NANC negative feedback in the absence of adrenergic support would result in destabilization of the normal negative feedback homeostatic controls. A further constrictor effect such as release of histamine and other constrictors would place the system under strain and lead to symptoms in those whose negative feedback homeostatic controls are relatively impaired.

The physiological role of the abundant β-adrenergic receptors in airways smooth muscle remains unclear but may relate to a fight or flight response. Certainly, bronchodilation is observed in the first few minutes of moderately intense exercise in both asthmatics (266) and, to a lesser extent, healthy subjects with bronchoconstriction in asthmatics typically not occurring until around 6–8 min of continuous exercise (65). This exercise related bronchodilation maybe related to the release of adrenaline or diminished vagal mediated parasympathetic tone (266). Their presence, despite the absence of a spinal adrenergic innervation, is fortuitous in that it permits the use of β-agonist therapy for the management of bronchoconstriction (with the caveat that tachyphalaxis in the absence of steroids occurs rapidly, perhaps due to the lack of neuronal innervation). If novel approach to preventing tachphylaxis were identified this may provide an effective alternative treatment. Interestingly the anticholinergic drug ipratropium bromide appears to produce greater dilatation in healthy individuals than the selective β-agonist salbutamol but in those with asthma the β-agonist is significantly more potent (267).

## Other Potential Factors

Amongst many suggestions as to factors that contribute to asthma is the possible contribution of changes in lung mechanics due factors such as “inflammation” or changes in blood flow. It is beyond the scope of this review to review these issues though it is worth reflecting again that inflammation *per se* as in CF, COPD etc. is not associated with significant changes in BHR, while pulmonary diseases such as obliterative bronchiolitis and cardiac defects such as a large VSD which result in high pulmonary blood volumes and flow are not typically associated with significant increases in BHR.

For many years, investigators have been seeking an epithelium-derived relaxing factor akin to the role played by nitric oxide in control of endothelial control but despite intense activity no such factor has been identified (268, 269). Others have suggested that the epithelium acts as an effective barrier to stimuli and that disruption of the epithelium is a key factor in the “hyper-reactivity” of asthma, a concept apparently supported by reports of characteristic epithelial damage in bronchial biopsies from asthmatic subjects. Larger, more detailed studies were unable to demonstrate significant differences in epithelial integrity when biopsies of asthmatics were compared with those from healthy individuals, though there was goblet cell hyperplasia (270–272).

## What Is the Mechanism Leading to Loss of Homeostatic Control?

This article proposes that asthma represents a loss of homeokinetic/homeostatic control in which a much greater variation in ASM length occurs than is consistent with normal function. In those with more severe asthma as can be seen in Figure 3 the baseline is also reduced (as reflected in a fall in maximal lung FEV1). This suggests a failure of the airways as whole to return to homeostatic point even for short periods. The manifestations of the underlying defect are variable, with *sensitivity* (which clearly is influenced by corticosteroids) influencing the likelihood of significant constriction, while *reactivity* determines the severity of airflow obstruction when constriction has been initiated, which may be attributable to greater shortening of the ASM units activated, or increased recruitment of ASM units producing more diffuse narrowing. The latter is probably largely determined at birth.

As noted above, it has long been debated whether there are fundamental changes in the function of ASM in asthmatic subjects in terms of contractility, such as an ability to generate more force, or greater shortening. Despite considerable efforts, there has been no convincing evidence for this. The reversible behavior of the ASM in response to corticosteroids over relatively short periods and over long periods when individuals “grow out” of their asthma suggests there is no intrinsic difference in ASM function *per se*.

Given that ASM changes its behavior toward the end of pregnancy from regular peristaltic waves sufficient to occlude the lumen moving distally (i.e., similar to gut from which it is derived), to one in which there is no significant constriction, it would appear that the ASM acquire a homeostatic mechanism that is aimed at preventing excessive constriction which could severely harm the organism in post-natal life. *Perturbation of this acquired state of homeostasis seems the most likely cause of asthma*. That this is a classical negative feedback control form of maintaining homeostasis is suggested by the observation that in healthy individuals there is a small but regular oscillation of airways caliber around a given mean value. One potential key observation is that ASM appear to oscillate about their mean largely independently of the other millions of cells within the airway and are not normally acting in a co-ordinated function such that the billions of potential combinations of narrowing and relaxing units result in a very small but clinically indiscernible small variations in overall airways caliber (205). These oscillations are likely to be controlled by ionic fluxes (193–196, 206, 273). In those with bronchoconstriction, this random oscillation appears to be perturbed with a net increase in units shortening with the random nature contributing to the inhomogeneity observed during asthma “attacks” (narrowing due to poor control or an exacerbation).

Quite how ASM tone is regulated to prevent excessive narrowing is unknown (274, 275). Possible mechanisms include (most likely first):

- Airways smooth muscle itself generates one or more autocrine negative feedback signals triggered by shortening (and lengthening) thus ensures ASM does not generate significant airways obstruction.
- The normal negative feedback homeostatic neural mechanisms is controlled centrally and ASM activity is driven by the parasympathetic cholinergic and NANC innervation in response to airways derived efferent signals.
- Non-ASM cells such as epithelial cells generate one or more negative feedback signals in response to compression, resulting from the mechanical stress generated during airways narrowing.
- Smooth muscle has an innate structural mechanism that limits significant shortening which becomes dysfunctional in asthmatic subjects.
- Post-natally ASM phasic contractions are random, non-co-ordinated. Given the vast number of ASM this ensures that there is no effective impact on lumen diameter. Bronchoconstriction occurs once a degree of co-ordination is re-established.
- The pacemaker driving peristaltic constriction *in utero* “switches off” toward the end of pregnancy but is “switched” back on to cause asthma and the pacemaker destabilizes the homeostatic control.
- ASM lose the ability to respond to an airways pacemaker but this response is restored in asthmatic subjects.
- ASM is a passive player and non-ASM structures within and/or surrounding the airways limit shortening and are altered by disease.

## Conclusion

Given that asthma appears to be a manifestation of a failure of post-natal homeostatic control of ASM there should be a shift in focus in asthma research toward understanding how stability is maintained in healthy airways and how, when this control is disrupted, it may be re-established. The focus may identify a single key missing feedback signal such as the loss of insulin in type 1 diabetes or, more likely, may be a result from a number of factors as observed in type 2 diabetes.

The observation that corticosteroids can largely or completely restore homeostasis and the recognition that natural resolution is relatively common, at least amongst those with relatively mild childhood asthma, would suggest that the aim should be to understand how normal homeostasis is maintained. As a result, we should finally be able to define the basic unifying underlying defect and ultimately be able to offer a cure.

## Author Contributions

ME and MA conceived and wrote the article collaboratively.

### Conflict of Interest

The authors declare that the research was conducted in the absence of any commercial or financial relationships that could be construed as a potential conflict of interest.

## Footnotes

^1^https://www.nationalasthma.org.au/living-with-asthma/resources/health-professionals/reports-and-statistics/the-hidden-cost-of-asthma-2015

^2^http://vintageadsandbooks.com/images/x/x088.jpg

^3^http://www.sign.ac.uk/pdf/sign101.pdf

^4^https://www.sign.ac.uk/sign-158-british-guideline-on-the-management-of-asthma.html

^5^https://ginasthma.org/reports/

^6^https://pathways.nice.org.uk/pathways/asthma#path=view%3A/pathways/asthma/asthma-overview.xml&content=view-node%3Anodes-diagnosis-and-monitoring
